# Prevalence of Endemic Pig-Associated Zoonoses in Southeast Asia: A Review of Findings from the Lao People's Democratic Republic

**DOI:** 10.4269/ajtmh.14-0551

**Published:** 2015-05-06

**Authors:** Anna L. Okello, Stephanie Burniston, James V. Conlan, Phouth Inthavong, Boualam Khamlome, Susan C. Welburn, Jeffrey Gilbert, John Allen, Stuart D. Blacksell

**Affiliations:** School of Biomedical Sciences, College of Medicine and Veterinary Medicine, The University of Edinburgh, Edinburgh, United Kingdom; School of Veterinary and Life Sciences, Murdoch University, Murdoch, Western Australia, Australia; National Animal Health Laboratory, Department of Livestock and Fisheries, Ministry of Agriculture, Vientiane, Lao People's Democratic Republic; Department of Hygiene and Prevention, Ministry of Health, Vientiane, Lao People's Democratic Republic; International Livestock Research Institute, Nairobi, Kenya; Australian Animal Health Laboratory (AAHL), CSIRO Animal, Food and Health Sciences, Geelong, Victoria, Australia; Mahidol-Oxford Tropical Medicine Research Unit, Faculty of Tropical Medicine, Mahidol University, Bangkok, Thailand; Centre for Tropical Medicine, Nuffield Department of Clinical Medicine, Churchill Hospital, Oxford, United Kingdom

## Abstract

The increasing intensification of pork production in southeast Asia necessitates an urgent requirement to better understand the dual impact of pig-associated zoonotic disease on both pig production and human health in the region. Sharing porous borders with five countries and representing many regional ethnicities and agricultural practices, the Lao People's Democratic Republic (Lao PDR) appears well placed to gauge the levels of pig-associated zoonoses circulating in the wider region. Despite this, little is known about the true impact of zoonotic pathogens such as leptospirosis, *Trichinella*, hepatitis E virus (HEV), Japanese encephalitis (JE), and *Taenia solium* on human health and livestock production in the country. A comprehensive review of the published prevalences of these five pig-associated zoonoses in Lao PDR has demonstrated that although suspicion remains high of their existence in pig reservoirs across the country, epidemiological data are scarce; only 31 epidemiological studies have been undertaken on these diseases in the past 25 years. A greater understanding of the zoonoses prevalence and subsequent risks associated with pork production in the southeast Asian region could help focus public health and food safety interventions at key points along the value chain, benefiting both livestock producers and the broader animal and human health systems in the region.

## Introduction

The growth in livestock production in recent years has raised concerns of the increasing risks of zoonotic disease transmission, with pig production in particular expanding in several regions of the world.^1^–^3^ Asia is a major global producer of pork, with southeast Asia and southern China currently comprising the majority of regional production.^4^ As pig production increases to meet growing demands, so too does the risk of disease transmission between pigs and humans; particularly in countries where comprehensive disease surveillance and response mechanisms are still being established. Pig-associated zoonoses previously described throughout parts of southeast Asia include *Taenia solium* cysticercosis, hepatitis E virus (HEV), *Streptococcus suis,* Japanese encephalitis (JE), and trichinellosis. Moreover, the role of pigs in the transmission of other significant zoonotic pathogens such as leptospirosis, brucellosis, and *Coxiella burnetii* (Q fever) are largely unknown. Production and trade implications from zoonoses such as *T. solium* cysticercosis, *S. suis*, brucellosis, and leptospirosis have the potential to further increase the socioeconomic impact of zoonotic disease through lost market opportunities.^5^ With pig consumption and production set to increase further across the globe, particularly in the southeast Asian region, the requirement to map the current knowledge of pig-associated zoonoses burdens, and highlight the gaps in our understanding regarding transmission and control, is becoming vitally important. This review summarizes the currently available published research on five pig-associated zoonoses considered to have important implications for animal and human health in Lao PDR: leptospirosis, trichinellosis, HEV, JE virus, and *T. solium*.

Lao PDR is a landlocked country in the Mekong Region bordered by China, Thailand, Vietnam, Cambodia, and Myanmar. Of the country's estimated 6.9 million people, only 32% are reported to reside in urban areas, with most relying heavily on the agricultural and forestry sectors for income.^6^–^8^ Classified as a low-middle-income country, pig production is an important defense against poverty; particularly in the northern provinces where up to 70% households raise pigs.^9^ Although some commercial pig operations can be found close to population centers, they are relatively small scale in comparison to those of neighboring countries.^10^,^11^ Despite this, there is evidence that pig production is growing in the country, increasing by 71% between 1998 and 2008, accompanied by an increase in output of pork products by 77% during the same period.^12^

## Methodology

The literature search was conducted in numerous databases including PubMed, Science Direct, Web of Science, BIOSIS, Journal of Citation Reports, CAB direct, and Google Scholar. A total of 430 abstracts were reviewed, with 152 full text reviews and 38 papers included in the final analysis. Identification of these publications within these databases occurred through using combinations of “(disease) AND Lao PDR” and “zoonotic disease AND Lao PDR,” where “disease” referred to the five diseases outlined in the introduction. Full articles were obtained if the initial abstract screening deemed the articles suitable for inclusion or exclusion under the following criteria:

### Inclusion criteria.

Articles from any journal published in English between 1990 and 2014 describing the incidence or prevalence of the five zoonoses in either humans or pigs in Lao PDR. Where available case reports, commentaries, editorials, grey literature, poster presentations and conference proceedings were also included in the analysis.

### Exclusion criteria.

Zoonotic diseases/syndromes in Lao PDR with the wrong disease agent or in species other than pigs or humans. Also excluded were genetic and molecular studies, development/evaluation of diagnostic techniques, vaccine studies, duplicated data, or articles written in a language other than English.

## Results

The current knowledge on the prevalence of endemic pig-associated zoonoses in Lao PDR is as follows.

### Leptospirosis.

Leptospirosis is an important reemerging zoonotic disease worldwide caused by the bacterial species *Leptospira interrogans*, which is subdivided into 24 serogroups and over 200 serovars based on the bacteria's outer envelope lipopolysaccharide (LPS). Risk factors for human infection appear mainly occupational, with veterinarians, abattoir workers, farmers, miners, and sewage workers all at increased risk of contracting the disease from infected animals through skin or mucous membrane contact.^13^,^14^ Environmental factors are also important, with waterborne transmission frequently reported during heavy rains and floods that expedite the leaching of *Leptospira* from the soil, resulting in major outbreaks.^13^ Although human to human transmission is not considered significant, ingestion of contaminated food or urine have also been cited as risk factors for disease transmission.^14^

Reports of human leptospirosis prevalence in Lao PDR have ranged from 3% to 49%. Two longitudinal hospital-based surveys in Vientiane found seroprevalences of 6.8% (2001–2004) and 14% (1993–2001) in patients admitted with acute jaundice or elevated liver enzymes during the years of study.^15^,^16^ A third multihospital survey undertaken in Vientiane from 1995 to 1996 found serological evidence of recent *Leptospira* infection in 21% and 8% of cases and controls, respectively.^17^ Further hospital-based surveys undertaken between 2008 and 2010 in Luang Namtha province in the north and Salavan province in the south of the country, found seroprevalences of 7% and 3%, respectively, in patients presenting with no obvious cause of fever.^18^

Community-level surveys across the country in 2006 reported antibody background prevalences of 49% (Vientiane), 45% (Luang Prabang), 37% (Borikhamxay), 19% (Champassack), and 24% (Khammouane province).^16^,^19^ For the majority of these studies however, no serogroup identification was carried out, apart from in Khammouane province where *Leptospira panama*, *Leptospira autumnalis*, *Leptospira hebdomadis*, and *Leptospira icterohemorrhagiae* accounted for 84% seropositive samples.^19^

Leptospirosis infection is less well documented in animals, with global prevalence reports ranging from 2% to 46% depending on the species.^14^,^20^ In Lao PDR, the seroprevalence of *L. icterohaemorrhagiae* serovar *hardjo-bovis* antibodies in cattle and buffalo has been estimated to be 3.1%,^21^ however no published data are available on the prevalence of leptospirosis in pigs, despite being reported in this species in neighboring Vietnam and Thailand.^22^–^25^ The impact of pig infection on vulnerable populations such as pig traders and butchers or those eating raw pork is unknown, however occupational exposure was suspected in one of the previous human studies in Lao PDR.^18^ In terms of pig production, leptospirosis can be subclinical, although significant reproductive losses including abortions, stillbirths, and weak piglets have been reported in other countries, including neighboring Vietnam and Thailand.^22^,^26^,^27^

### *Trichinella*.

Trichinellosis is one of the most widely distributed parasitic zoonoses worldwide, with an estimated 11 million people at risk of infection.^28^ Caused by one of eight *Trichinella* nematode parasites, three have been implicated in outbreaks of human disease in southeast Asia to date: *Trichinella spiralis*, *Trichinella pseudospiralis*, and *Trichinella papuae*.^12^,^29^,^30^ *Trichinella* infection mainly occurs through the consumption of raw or undercooked meat—including pork—containing nematode larvae^31^; analysis of 50 years of epidemiological reports from China showed that the consumption of raw or undercooked pork was implicated in 94.3% human trichinellosis cases nationwide during this time.^32^ Unregulated or home slaughter of pigs, particularly in areas where free-ranging pigs are in contact with wild animals, remains a high-risk factor for transmission.^33^ Although no available data could be found on the prevalence of *Trichinella* in wild animal populations in the region, the consumption of wild boar has previously been implicated in outbreaks of *T. papuae* in Thailand.^34^,^35^ Furthermore, the role of rats in *Trichinella* transmission requires further investigation^36^,^37^; if found to be significant, this would encourage rat control interventions to simultaneously address *Trichinella* and leptospirosis in communities at risk for both.

Traditional ceremonial events and large gatherings such as weddings and New Year celebrations have been associated with recent *Trichinella* outbreaks in southeast Asian countries including China, Thailand, Vietnam, and Lao PDR.^31^,^34^,^35^,^38^–^42^ In one post-outbreak study in Vietnam, 30% participants demonstrated antibodies to *Trichinella* after consuming raw or undercooked wild boar at a Vietnamese lunar year celebration^41^; large outbreaks have also been reported in neighboring Thailand as a consequence of eating undercooked pork at festivals and parties.^35^ Transmission also occurs through food preparation; most households in Lao PDR use the same utensils for both cooked and raw food.^43^ Although socioeconomic status has been negatively linked to parasite infection in many cases, increased economic status may also result in an increased risk of acquiring infection because of increased meat consumption.^31^,^44^ The type of pig production enterprise has been found to impact on disease status; free-ranging pig systems common to smallholder communities in Lao PDR and elsewhere in the region are associated with an increased risk of infection where pigs forage and feed on *Trichinella*-infected food scraps.^32^,^36^,^45^,^46^

*Trichinella* is endemic in Lao PDR^14^,^47^; with pork products implicated in all four published outbreak reports.^31^,^33^,^39^,^43^ The majority of reported outbreaks occurred in the ethnically diverse northern and central regions, where raw or undercooked pork appears to be consumed relatively frequently.^39^,^47^ The largest documented outbreak in Lao PDR took place in the northern province of Oudomxay in 2005, involving an estimated 650 patients.^39^ A smaller outbreak of 21 confirmed cases occurred in central Borikhamxay province during Laos New Year in 2004.^31^ A cross-sectional survey of 1419 individuals in northern Lao PDR in 2009 via enzyme-linked immunosorbent assay (ELISA) seroprevalence testing followed by the gold standard Western blot on a subset of positives, determined that up to 20% of the population were exposed to *Trichinella* larvae.^47^ Males and people of the Lao-Tai ethnic group were found to be at greatest risk of having antibodies detected; the odds of being seropositive were also spatially heterogeneous, with an increased risk of *Trichinella* antibodies with increasing age.^47^

Limited data exist on the prevalence and species of *Trichinella* in pigs in Lao PDR. In northern Lao PDR, *Trichinella* infection in pigs (as determined by artificial muscle digestion) was found to be 2.1% (15/728) across four provinces, highest in Xieng Khouang province (4.8%).^47^ DNA extracted from 13/15 larvae were found to be *T. spiralis,* similar to a smaller survey of pig meat in Oudomxay province in 2005.^47^ Although limited serological data exist in Lao PDR, another recent survey across two geographically distinct provinces identified a crude *Trichinella* seroprevalence of 13.7%.^48^ In northwestern Vietnam, Vu Thi and others^49^ reported a seroprevalence of 19.9% in domestic free-range pigs where previous human trichinellosis had occurred; *T. spiralis* larvae were identified via PCR in 14.5% of these seropositive animals. In neighboring China, surveys of pigs by direct diagnostic tests (microscopy or artificial muscle digestion) to detect *Trichinella* infection have demonstrated a spatially heterogeneous distribution, with prevalence in pigs ranging from < 4% in Yunnan and Guangxi provinces (southern China) to 34% in a county of Henan province in central China.^32^

### Hepatitis E Virus.

HEV is an RNA virus phylogenetically classified into four genotypes (1–4). Although genotypes 1 and 2 are exclusively human pathogens, a zoonotic reservoir for genotypes 3 and 4 has been identified through the detection of anti-HEV antibodies in several animals, including pigs where it is often referred to as “swine HEV.”^50^–^53^ In southeast Asia, the disease predominantly affects those living in rural areas, with occupational exposure and smallholder pig production likely to incur a greater risk of infection.^54^–^57^ Similar to other pig-associated zoonoses, an important risk factor in Lao PDR remains the consumption of raw or undercooked pork in conjunction with free-ranging production systems and poor sanitation.^58^,^59^ Although reported outbreaks in Lao PDR have all been food borne, contamination of water resources has been implicated with recurrent HEV epidemics in other parts of southeast Asia.^60^–^62^ This has an implication for villages whose water sources originate in local rivers and streams, especially after wet-season flooding; further research is required to assess the impact of seasonality on HEV transmission, particularly of the zoonotic genotypes.^17^ HEV transmission from pigs to humans can occur indirectly via the fecal-oral route, for example through the ingestion of feed or water contaminated with pig faeces,^62^ or via direct foodborne transmission after consuming meat or viscera from infected animals.^56^ The relatively common practices in Lao PDR of using pig manure as fertilizer for vegetable gardens and raising pigs over fish ponds are therefore potentially significant sources of human infection.^58^,^59^

The extent of HEV infection in humans in Lao PDR is not fully understood, particularly when compared with other known hepatitides and/or causes of acute jaundice such as Hepatitis A.^17^ Reported HEV prevalences have ranged from 1.6% in patients presenting with acute jaundice or hepatitis at Mahosot hospital in Vientiane,^15^ whereas an earlier multihospital study in Vientiane found no significant difference between HEV IgM antibodies in presenting acute jaundice cases (16%) and controls (17%).^17^ This latter finding suggests that although the level of exposure to HEV may be quite high in the country, the disease does not commonly manifest clinically as acute jaundice. The exception is the clinical impact of HEV on pregnant women, where reported mortality rates as high as 28% in some countries^52^ necessitate the requirement to fully establish the role of pigs in disease transmission.^15^,^60^,^63^

Reported HEV prevalences from pigs in Lao PDR have ranged from 8.8% to 85.7%, with particularly high prevalences found in the north of the country.^59^,^63^,^64^ One study has also identified pigs in Lao PDR to be carriers of the zoonotic genotype 4,^63^ reported elsewhere in Asia including China, Taiwan, Japan, and Vietnam.^50^,^65^ Conlan and others reported a probable age and temporal (wet versus dry season) linkage to HEV infection in pigs, finding that seroprevalence appeared the highest in young animals at the beginning of the wet season.^64^

### Japanese Encephalitis Virus.

The vector-borne flavivirus JE is the leading cause of viral encephalitis in Asia, disproportionately impacting children aged 1–15 years in endemic areas.^66^ JE transmission is a classic manifestation of a disease issue at the human-animal-environment interface; the mosquito vector breeds in rice fields, maintaining a zoonotic cycle of transmission between humans and ardeid wading birds and/or pigs, with the proximity of human households to irrigated rice fields a known risk factor for disease transmission.^66^–^68^ A survey in Khammouane province (south central Laos) also found the quality of house construction to be an important risk factor, due to its impact on the level of occupant exposure to infected mosquitoes.^69^ There is a significant risk of JE transmission in a number of areas in Lao PDR where smallholder pig husbandry, rice production systems, and poverty coexist.

A review of reported JE prevalence in humans in Lao PDR has identified ranges between 0% and 11% in a hospital-based survey in Vientiane of children 15 years of age and under.^70^ Other hospital-based surveys in the country have found seroprevalences of up to 75% in Khammouane province, 7% in Luang Namtha, and 4% in Salavan province.^18^ Various community level surveys have also found a range of seroprevalences, including 10% and 80% in Khammouane province and Vientiane Municipality, respectively,^71^,^72^ and 2.1% in a large-scale serosurvey in Vientiane city.^73^ In a community level survey in the Nakai Plateau, a seroprevalence of 40% was reported, identifying 10% of these cases as JE once the dengue cross-reactions were removed.^74^ There is further serological evidence of the virus throughout the country, however diagnosis is complicated by cross-reactivity of JE with other endemic flaviviruses such as dengue virus.^75^ Limited data exist for JE prevalence in pigs in Lao PDR, however a recent survey in the north showed hyperendemicity, with prevalence ranging from 59% to 90% in four northern provinces with peak transmission, as demonstrated by IgM detection, occurring in the wet season.^64^

### *T. solium* cysticercosis and taeniasis.

Humans are both the definitive and accidental dead-end intermediate hosts of the *T. solium* parasite; able to be infected with both the adult (tapeworm) and larval (cystic) forms of disease. Encystment of *T. solium* larvae in the human central nervous system results in a condition known as neurocysticercosis (NCC), the leading cause of acquired epilepsy in the developing world.^76^ Although the relationship between human cysticercosis infection and epilepsy in southeast Asia has not been fully elucidated, NCC is likely an important cause of epilepsy in areas with a high human taeniasis prevalence.^77^

Given the relatively complicated *T. solium* transmission cycle, there are a number of behavioral factors that can increase the likelihood of human ingestion of viable cysts from infected pork meat and the ingestion of viable tapeworm eggs by both pigs and humans from contaminated environments. Risk factors include poverty, poor hygiene and sanitation, open defecation, free roaming pigs, consumption of raw or improperly cooked pork, and informal slaughter and trade leading to poor control of infected carcasses.^9^,^78^ Even in urban areas with improved hygiene and sanitation, the consumption of raw pork and uncooked vegetables, use of human feces as fertilizer, and problems of waste and wastewater management all promote transmission.^9^,^78^ For example, a case-control study by Tran and others^79^ found a significant correlation between the use of human feces as fertilizer and epilepsy in Lao PDR, rendering this practice an important risk factor in the country. Risk factors for infection in pigs include access to human feces through indiscriminate disposal of feces around households.^45^,^80^ Coexistence of other *Taenia* species such as *Taenia asiatica* and *Taenia hydatigena* (with the definitive host being humans and dogs, respectively) is suspected to be protective in pigs, reducing the ability of *T. solium* to establish in the pig population, and thereby decreasing the risk to the human population.^81^,^82^

In Lao PDR, reports of human *Taenia* infections (species not determined) range from 0% to 17%, with a high degree of spatial and temporal variation.^81^ However, given the low specificity of existing diagnostic methods in pigs and humans, the prevalence of the relatively nonpathogenic *T. asiatica* and *Taenia saginata* tapeworm species are important to specify in the Asian context.^9^,^31^,^44^,^83^–^90^ Recently, an unusually high *T. solium* antigen prevalence of 26.1%, demonstrating active human taeniasis infection in positive individuals, was discovered in a secluded minority ethnic group in northern Lao PDR, suggesting that cultural practices around sacrificial slaughter could play an important role in some areas.^91^

Data regarding the prevalence and incidence of human cysticercosis in Lao PDR are also limited,^78^ with only a small number of individual NCC cases definitively diagnosed via computed tomography (CT) scan in the country.^92^ Two serosurveys using ELISA diagnostics found anti-cysticercosis antibody prevalences of 4.8% in Vientiane province^79^ and 2.2% in 1306 samples collected from four northern provinces.^81^ Although some national estimates of antibody seroprevalence are as high as 10%,^93^ a 2011 study using the highly sensitive and specific enzyme-linked immunoelectrotransfer blot (EITB)–strip test found prevalences of over 60% in some parts of the country, suggesting hyperendemic “pockets” of cysticercosis likely exist in Lao PDR.^91^

Porcine cysticercosis has also been reported in Lao PDR, however prevalence data are rarely available, with swine serology complicated by cross-reactivity of available serological diagnostics with benign *T. hydatigena* and *T. asiatica* infections.^78^,^90^ Slaughterhouse inspection of 590 pig carcasses in three provinces in the north of the country revealed 0.8% pigs to be heavily infected with cysts consistent with *T. solium* morphology, with *T. hydatigena* cysts reported in 22.4% pigs.^81^ Aside from ensuring the maintenance of *T. solium* in susceptible human populations, *T. solium* cysts in pigs can also result in widespread economic losses.^5^,^94^ The requirement for more readily available, sensitive, and specific diagnostic tools for detecting and differentiating porcine *Taenia* species in the Asian context, as well as understanding the true prevalence and burden of NCC in the region, is urgently required.^95^

## Discussion and Conclusion

The current knowledge regarding the impact of endemic pig-associated zoonoses to both human health and pig production in Lao PDR is summarized as follows.

To date, regional animal health programs in southeast Asia have placed a significant focus on trade-impacting transboundary animal diseases (TADs) such as foot-and-mouth disease (FMD) and classical swine fever (CSF). However, the rapid changes occurring in the demand for pork products in the region highlights a further requirement to critically assess the risk of pig-associated zoonotic diseases also circulating within these systems, under broader food and environmental safety narratives.

Despite its importance, we found only 31 studies—including review papers and nonspecific investigations of jaundice and fever—have been published from Lao PDR since 1990 on the five focus zoonotic diseases (Table 1). Of these, 13 were classified as *T. solium* studies, however many of these were undertaken as part of broader human investigations on soil-transmitted helminths where the *Taenia* species was not identified, therefore were not directly reflective of a *T. solium* research focus. Furthermore, it should be noted that no published studies currently exist on the potential significance of zoonoses such as brucellosis and *C. burnetii* (Q fever) in pig-rearing systems in Lao PDR. A third observation was that the majority of published data, particularly for leptospirosis, HEV, and JE, were from hospital-based surveys, often from the capital city Vientiane. Although this is valuable information, it must be remembered that patients presenting at urban hospitals may not reflect the true demographic of zoonoses sufferers in a country, given the propensity for zoonoses to cluster in high risk, often rural areas where accessibility to health services and appropriate diagnostic facilities is frequently lacking.^105^ The authors recommend more ground level surveys to be undertaken at the provincial and district levels across the country, particularly where high underreporting is suspected as a result of physical, social, or economic barriers to health care.

For leptospirosis, four human studies have been conducted in Lao PDR to date, with antibody prevalences from 3% to 49% reported through a combination of hospital and district level surveys. However, the void of available information on porcine leptospirosis in the country means the role of pigs—if any—on leptospirosis transmission is still unknown in Lao PDR. For HEV, five published studies have reported prevalence ranges of 1.6–17% and 8–85% in humans and pigs, respectively. The confirmation of zoonotic genotype 4 in pigs in Lao PDR, coupled with the common practice of known risk factors and a high prevalence confirmed in several pig populations, suggests that pigs are indeed a significant source of human exposure in the country. Given the most severe clinical manifestations of HEV occurs in pregnant women, the authors recommend that HEV be considered in ongoing maternal and child health programs in Lao PDR, with women of childbearing age alerted to the risks posed by the unsafe handling and consumption of pork products. Evidence of JE in the country, with antibody prevalences as high as 80% in humans and 90% in pigs in some areas, suggests vigilance toward mosquitoes should also be maintained, particularly in rural areas where smallholder pig-rearing systems simultaneously exist alongside rice cultivation.

The consumption of raw pork and pork products is relatively common in Lao PDR, particularly for certain ethnic groups, reflected in several epidemiological studies that have shown exposure to *Trichinella* and *T. solium* to be higher in certain ethnicities. Outside documented outbreaks in Lao PDR, only one cross-sectional human study has been published, demonstrating that up to 20% individuals have been exposed to *Trichinella* parasites in the north of the country. A more recent presentation to the Kunming EcoHealth Conference in 2012 found a crude seroprevalence of 47.3%, however the adjusted prevalences have not yet been published. Only one study has been published to date on the levels of *Trichinella* in pork, where reported levels of 2.1% were recorded from a series of slaughterhouse surveys in the north of the country. Similarly for *T. solium*, the currently available information is lacking, however the discovery of *T. solium* taeniasis antigens and cysticercosis antibody prevalences as high as 26.1% and > 60%, respectively, in some communities highlights the urgent requirement to develop a robust methodology for detecting these hyperendemic clusters of *T. solium* in the country.

To overcome the suspected underreporting of pig-associated zoonoses in both Lao PDR and neighboring countries in the region, further development of robust porcine disease surveillance and monitoring systems—built where possible around existing supply chains and cross-border trading points—is recommended. The benefits of good surveillance extend beyond the impact on endemic zoonoses, via simultaneously promoting the early detection of pig zoonoses with pandemic potential such as swine influenza subtypes and henipah viruses. Identifying and understanding the zoonotic disease risks of pig production, slaughter, and consumption should be integral to any programmatic intervention aimed at improving the pork value chains in southeast Asia. Aside from the important public good aspects of zoonoses control, knowing the circulating disease risks will also improve the cost-effectiveness of interventions, through targeting several diseases at once in known co-endemic areas. Possible examples include community-based health strategies that incorporate *T. solium* and *Trichinella* into existing hygiene and sanitation interventions or slaughterhouse improvements that address occupational hazards such as leptospirosis and HEV in addition to foodborne diseases. A transdisciplinary one health approach toward pig-associated zoonotic disease, incorporating both epidemiological and social research approaches, is highly recommended.

## Figures and Tables

**Table 1:** Summary of reviewed literature and reported prevalences

Organism	No. of studies specific to Lao PDR*	Reference list—Lao PDR*	Further references (broader Asia region)*
*Leptospira*	4	• Syhavong and others^15^	• Laras and others^16^ (southeast Asia)
		• Bounlu and others^17^	• Pappas and others^96^ (global)
		• Mayxay and others^18^	• Bordier and Roger^97^ (global)
		• Kawaguchi and others^19^	• Victoriano and others^98^ (Asia and Pacific)
			• Myint and others^99^ (Thailand)
			• Seng and others^100^ (Cambodia)
			• Hartskeerl^101^ (Indonesia)
*Trichinella*	5	• Sayasone and others^31^	• Bordier and Roger^97^ (global)
		• Taybouavone and others^33^	• Takahashi and others^38^ (Asia and Pacific)
		• Barennes and others^39^	
		• Suwansrinon and others^43^	
		• Conlan and others^47^	
HEV	5	• Syhavong and others^15^	• Bordier and Roger^97^ (global)
		• Bounlu and others^17^	• Corwin and others^60^ (southeast Asia)
		• Blacksell and others^59^	• Corwin and others^61^ (Indonesia)
		• Conlan and others^63^	
		• Conlan and others^64^	
JE virus	5	• Bounlu and others^70^	• Bordier and Roger^97^ (global)
		• Makino and others^71^	• Erlanger and others^104^ (global)
		• Vongxay^72^	
		• Vallée and others^73^	
		• Hiscox and others^74^	
*Taenia solium*	13†	• Conlan and others^9^	• Bordier and Roger^97^ (global)
		• Tran and others^79^	• Montresor and Palmer^102^ (global)
		• Conlan and others^81^	• Willingham^95^ (southeast Asia)
		• Sayasone and others^83^	• Rajshekhar and others^103^ (Asia)
		• Chai and Hongvanthong^84^	• Dorny and others^78^ (Cambodia, Lao PDR, and Vietnam)
		• Rim and others^85^	
		• Phongluxa and others^86^	
		• Chai and others^87^	
		• Chai and others^88^	
		• Vannachone and others^89^	
		• Jeon and others^90^	
		• Okello and others^91^	
		• Jeon and others^92^	

HEV = hepatitis E virus; JE = Japanese encephalitis; Lao PDR = People's Democratic Republic.

* Matching inclusion and exclusion criteria.

† Based on reports of *Taenia* spp.; species identification not performed in all surveys.
